# Drug resistance after cessation of efavirenz-based antiretroviral treatment started in pregnancy

**DOI:** 10.4102/sajhivmed.v21i1.1023

**Published:** 2020-01-27

**Authors:** Globahan Ajibola, Christopher Rowley, Dorcas Maruapula, Jean Leidner, Kara Bennett, Kathleen Powis, Roger L. Shapiro, Shahin Lockman

**Affiliations:** 1 Botswana Harvard T.H. Chan School of Public Health AIDS Initiative Partnership, Gaborone, Botswana; 2 Department of Immunology and Infectious Diseases, Harvard T.H. Chan School of Public Health, Boston, United States; 3 Beth Israel Deaconess Medical Center, Boston, United States; 4 Goodtables Data Consulting, LLC., Norman, United States; 5 Bennett Statistical Consulting, Inc., Ballston Lake, United States; 6 Department of Medicine, Division of General Internal Medicine, Massachusetts General Hospital, Boston, United States; 7 Department of Pediatrics and Pediatric Surgery, Massachusetts General Hospital, Boston, United States; 8 Division of Infectious Disease, Brigham and Women’s Hospital, Boston, United States

**Keywords:** drug resistance, resistance mutations, HIV, antiretroviral treatment, Botswana

## Abstract

**Background:**

To reduce risk of antiretroviral resistance when stopping efavirenz (EFV)-based antiretroviral treatment (ART), staggered discontinuation of antiretrovirals (an NRTI tail) is recommended. However, no data directly support this recommendation.

**Objectives:**

We evaluated the prevalence of HIV drug resistance mutations in pregnant women living with HIV who stopped efavirenz (EFV)/emtricitabine (FTC)/tenofovir disoproxil fumarate (TDF) postpartum.

**Method:**

In accordance with the prevailing Botswana HIV guidelines at the time, women with pre-treatment CD4 > 350 cells/mm^3^, initiated EFV/FTC/TDF in pregnancy and stopped ART at 6 weeks postpartum if formula feeding, or 6 weeks after weaning. A 7-day tail of FTC/TDF was recommended per Botswana guidelines. HIV-1 RNA and genotypic resistance testing (bulk sequencing) were performed on samples obtained 4–6 weeks after stopping EFV. Stanford HIV Drug Resistance Database was used to identify major mutations.

**Results:**

From April 2014 to May 2015, 74 women who had stopped EFV/FTC/TDF enrolled, with median nadir CD4 of 571 cells/mm^3^. The median time from cessation of EFV to sample draw for genotyping was 5 weeks (range: 3–13 weeks). Thirty-two (43%) women received a 1-week tail of FTC/TDF after stopping EFV. HIV-1 RNA was available from delivery in 70 (95%) women, 58 (83%) of whom had undetectable delivery HIV-1 RNA (< 40 copies/mL). HIV-1 RNA was available for 71 women at the time of genotyping, 45 (63%) of whom had HIV-1 RNA < 40 copies/mL. Thirty-five (47%) of 74 samples yielded a genotype result, and four (11%) had a major drug resistance mutation: two with K103N and two with V106M. All four resistance mutations occurred among women who did not receive an FTC/TDF tail (4/42, 10%), whereas no mutations occurred among 18 genotyped women who had received a 1-week FTC/TDF tail (*p* = 0.053).

**Conclusions:**

Viral rebound was slow following cessation of EFV/FTC/TDF in the postpartum period. Use of an FTC/TDF tail after stopping EFV was associated with the lower prevalence of subsequent NNRTI drug resistance mutation.

## Introduction

Cessation of antiretrovirals raises concerns about the potential for the development of drug-resistant viral strains, particularly for non-nucleoside reverse transcriptase inhibitors (NNRTIs). Non-nucleoside reverse transcriptase inhibitors, such as efavirenz (EFV), have long half-lives and a low barrier to the development of drug resistance. When used in combination with other classes of drugs such as nucleoside reverse transcriptase inhibitors (NRTIs), staggered discontinuation using a 4–7-day ‘tail’ of the NRTI backbone when stopping NNRTI-based antiretroviral treatment (ART) is advised.^1^ This is believed to mitigate the unintended period of monotherapy with NNRTIs that ensues because of the long half-life of most drugs in this class, thus reducing the risk of developing drug resistance.^2,3^ There are, however, very limited data to directly support this recommendation for a tenofovir disoproxil fumarate (TDF)/emtricitabine (FTC) ‘tail’: following cessation of EFV. Of note, pharmacokinetic variability (linked genetic factor – CYP2B6) in EFV metabolism between individuals has been observed, and EFV levels may be higher in persons of African descent.^4^ These higher drug levels may also impact risk of drug resistance when stopping EFV-based ART.

Prior to 2015, both the World Health Organization (WHO) and the Botswana programme for the prevention of mother-to-child transmission (PMTCT) recommended that pregnant women living with HIV with a CD4 cell count > 350 cells/mm^3^ without a WHO stage 3 or 4 illness take three-drug ART in pregnancy, but discontinue the ART postpartum or upon breastfeeding cessation (option B). Although both WHO and Botswana subsequently modified guidelines to recommend lifelong ART regardless of CD4 cell count or disease stage (option B+), the prior guidance for pregnant women, as well as the inconsistent application of an NRTI tail at the time of EFV cessation, offers an opportunity to evaluate the development of resistance and the performance of a TDF/FTC tail among women stopping an EFV/TDF/FTC regimen. Because this ART regimen remains widely used throughout the world, the natural experiment afforded by prior PMTCT guidelines is applicable to our understanding of the risk of cessation of NNRTI-based regimens generally.

## Methods

### Study population

Between April 2014 and May 2015, we enrolled a subset of pregnant women living with HIV participating in a trial of infant cotrimoxazole prophylaxis in Botswana (the ‘Mpepu’ study) into this resistance sub-study. Mpepu was a double-blinded randomised controlled trial designed to assess the efficacy and safety of infant cotrimoxazole prophylaxis versus placebo used daily from as early as 14 days of life through 15 months among HIV-exposed uninfected infants in Botswana.^5^

Women were eligible for this sub-study of drug resistance following cessation of EFV/FTC/TDF if they were living with HIV, had pre-treatment CD4 > 350 cells/mm^3^ without evidence of WHO stage 3 or 4 disease, initiated EFV/FTC/TDF in pregnancy and stopped ART postpartum according to Botswana guidelines at the time (at 4–6 weeks postpartum if formula feeding, or 6 weeks after weaning if breastfeeding). Per Botswana national HIV treatment guidelines, a 7-day tail of TDF/FTC after cessation of EFV was recommended. However, decisions regarding receipt and cessation of maternal ART occurred at government clinics (rather than study clinics), and the 7-day tail was inconsistently applied at the time of ART cessation.

### Data collection and laboratory testing

Women eligible to participate in this sub-study were identified in the Mpepu study and scheduled for a clinic visit 4–6 weeks after ART cessation. Interested and eligible women consented to participate in the study and provided data (from self-report and medical records) on PMTCT regimen received; dates on which ART and individual antiretrovirals were started and stopped as well as provided blood specimens for drug resistance and HIV-1 RNA testing. HIV RNA results at the point of enrolment into the Mpepu study, and documented nadir CD4 counts, were retrieved and served as baseline values.

Prior to drug resistance testing, we first determined HIV-1 RNA viral load levels with the Abbott m2000sp/rt machine (limit of detection 40 copies/mL). Where HIV-1 RNA was detected, it was extracted using an automated validated technique.^6^ We then performed reverse transcription using in-house one-step RT-PCR technique.^7^ Polymerase chain reaction (PCR) products were then purified^8^ and sequenced.^9^ Consensus sequences were generated, and the Stanford HIV Drug Resistance Database was used to identify all drug resistance mutations in the protease and reverse transcriptase coding regions.

### Statistical methods

Statistical Package for Social Sciences (SPSS) version 25 was used to perform statistical analyses, which were generally descriptive in nature; a two-sided Fisher’s exact test was used to evaluate for differences in the proportion of drug resistance by the approach to ART cessation, either with a 7-day TDF/FTC tail or with abrupt cessation of all three drugs. The small number of women with drug resistance mutations precluded more detailed analysis of predictors of drug resistance.

### Ethical onsiderations

The Botswana Health Research Development Committee and the Office of Human Research Administration at Harvard T. H. Chan School of Public Health approved this drug resistance sub-study (HRDC 00732 and IRB13-2772), and women provided written informed consent for participation.

## Results

### Baseline characteristics

Ninety women enrolled, 74 of whom discontinued EFV/FTC/TDF postpartum and had samples collected for genetic resistance testing and are included in this analysis. Sixteen of 90 women not included in this analysis had CD4 < 350 cell/mm^3^ post-delivery and had to continue ART for their own health per Botswana national protocol at the time. The median time from cessation of EFV (whether or not a tail period occurred) to time sample was collected for resistance testing was 5 weeks (range: 3–13 weeks). Median age at enrolment was 29 years (range: 20–45) with median nadir CD4 count of 571 cells/mm^3^ (range: 361–1236). Forty-seven (64%) women had no previous exposure to antiretrovirals, 25 (34%) reported previous exposure to antiretrovirals for PMTCT purposes in a prior pregnancy and 2 (2%) were previously exposed to EFV/FTC/TDF but stopped prior to conception and then re-started EFV/FTC/TDF as three-drug prophylaxis for the index pregnancy. Thirty-two (43%) stopped ART with the recommended 1-week tail of TDF/FTC after cessation of EFV and 42 (57%) stopped all treatment at once. Of 70 women with viral load results at enrolment into the Mpepu study, 58 (83%) had HIV-1 RNA < 40 copies/mL. At 4–6 weeks post-EFV/FTC/TDF cessation, 44 (62%) of 71 women with viral load result had HIV-1 RNA < 40 copies/mL. Thirty-three (47%) women had HIV-1 RNA < 40 copies/mL at delivery and at the time of sample draw for genotyping (post-ARV cessation).

### Mutations

Thirty-five (47%) of 74 samples were successfully sequenced, with 4 (11%) of 35 having a major drug resistance mutation: 2 with K103N and 2 with V106M (Table 1). All four resistance mutations occurred among women who did not receive a TDF/FTC tail (4/42, 10%), whereas no mutations occurred among 18 genotyped women who had received a TDF/FTC tail (*p* = 0.053). Eighteen (58%) of the 31 women who had a genotype result and no drug resistance mutation took a TDF/FTC tail. Of 39 samples that could not be sequenced, 37 (95%) had HIV-1 RNA < 40 copies/mL in the sample being tested.

**TABLE 1:** Characteristics of women with successful sequencing post-antiretroviral treatment cessation.

Age (years)	Prior use of ART for own health	Prior use of ART for PMTCT	Received TDF/FTC tail	Duration of ART use (weeks)	Nadir CD4 (cell/UI)	Baseline VL (copies/mL)	Post ART cessation VL (copies/mL)	Mutation
27	No	Yes	Yes	31.6	518	< 40	42 166	E138A
27	No	No	Yes	38.4	570	< 40	< 40	E138A
23	No	No	Yes	40.7	710	< 40	13 120	K103N
25	No	No	Yes	10.1	572	< 40	1719	K103N
35	No	No	No	54.5	772	< 40	18 259	None
40	No	Yes	Yes	31.2	442	< 40	2658	None
36	No	Yes	No	20.4	963	< 40	< 40	None
25	No	No	No	20.2	611	< 40	857	None
35	No	No	No	11.4	430	< 40	-	None
25	No	No	Yes	15.8	575	< 40	33 906	None
24	No	No	Yes	25.2	496	< 40	5688	None
26	No	Yes	No	25.6	460	< 40	2342	None
27	No	Yes	Yes	14.0	908	< 40	1805	None
29	No	No	Yes	23.1	436	58	379	None
42	No	No	Yes	27.8	533	< 40	< 40	None
40	No	No	No	13.5	361	52	250	None
33	Yes	No	Yes	10.8	796	< 40	< 40	None
30	No	No	No	21.1	616	-	9764	None
26	No	No	No	24.9	1037	< 40	554	None
27	No	No	Yes	16.1	601	< 40	3648	None
40	No	No	Yes	24.6	363	< 40	33 432	None
27	No	No	No	81.6	673	< 40	196 735	None
24	No	No	Yes	39.2	800	< 40	< 40	None
31	Yes	No	No	36.7	1236	< 40	-	None
31	No	Yes	Yes	11.0	589	125	< 40	None
29	No	No	No	18.5	715	< 40	2261	None
26	No	Yes	No	96.2	612	<40	< 40	None
41	No	No	No	82.1	840	-	2229	None
35	No	Yes	No	45.8	960	< 40	4536	None
40	No	Yes	No	26.8	679	< 40	972	None
28	No	No	No	21.9	436	< 40	40 709	None
26	No	No	No	23.2	397	< 40	309	None
24	No	No	No	26.5	468	52	< 40	None
20	No	No	Yes	22.4	384	45	68†	V106M
33	No	Yes‡	Yes	23.8	468	< 40	145 392	V106M

ART, antiretroviral treatment; FTC, emtricitabine; TDF, tenofovir disoproxil fumarate; VL, viral load.

† , Genotypes were performed independent of the knowledge of the participants’ viral load; therefore, verification of the viral load on individual plasma sample was not performed.

‡ , Previous exposure to zidovudine for prevention of mother-to-child transmission (PMTCT).

## Discussion

A small proportion of women stopping EFV/FTC/TDF had a major NNRTI resistance mutation detected in plasma taken a little more than a month after stopping treatment. Among women receiving a 7-day tail of TDF/FTC after stopping EFV where genetic resistance testing was able to be performed, none had detectable mutations. Our findings thus support the recommended staggered discontinuation of antiretrovirals when an EFV/FTC/TDF regimen is being stopped. Our data support prior studies that evaluated the use of an NRTI tail when discontinuing EFV-based ART.

Our observation of prolonged viral suppression < 40 copies/mL for a median of 5 weeks in 62% of women post-ART cessation was similar to findings from other studies which have demonstrated that it is not unusual for viral suppression to persist for weeks after stopping ART.^10^ We believe this could be because of low pre-ART viral loads in our population (because of women with less advanced disease starting ART in pregnancy).

Our study had several limitations. Firstly, our results are based on a small number of observations making generalisability challenging. Secondly, we did not report on minor drug resistance variants and, finally, more than half (63%) of women tested at a median time of 5 weeks from EFV cessation were still virally suppressed, which may suggest that we might have tested for resistance strain development early. Thirdly, we could not determine why the 7-day tail was inconsistently applied at the time of ART cessation and could therefore have missed evaluating possible confounders related to this factor.

## Conclusion

Although guidelines no longer recommend discontinuation of ART after pregnancy, our study has general applicability for those requiring permanent or temporary treatment discontinuation when receiving an EFV-based regimen, informing the risk of mutation emergence in the setting of abrupt three-drug discontinuation versus discontinuing with a TDF/FTC tail. Furthermore, despite the shift to the use of newer drugs such as dolutegravir for which there is less concern about the emergence of resistance than with NNRTIs, our data are relevant to millions of individuals still on EFV-based ART (and are of particular clinical importance in regions that have access to only a limited number of antiretroviral drugs and that are also generally unable to routinely assess for drug resistance mutations before initiating a new ART regimen).

Based upon our results, we conclude that an TDF/FTC tail may help prevent selection of major NNRTI drug resistance mutations that can occur with the abrupt discontinuation of an EFV/FTC/TDF ART regimen.
